# Future Climate CO_2_ Levels Mitigate Stress Impact on Plants: Increased Defense or Decreased Challenge?

**DOI:** 10.3389/fpls.2016.00556

**Published:** 2016-05-02

**Authors:** Hamada AbdElgawad, Gaurav Zinta, Gerrit T. S. Beemster, Ivan A. Janssens, Han Asard

**Affiliations:** ^1^Integrated Molecular Plant Physiology Research, Department of Biology, University of AntwerpAntwerp, Belgium; ^2^Faculty of Science, Department of Botany, University of Beni-SuefBeni-Suef, Egypt; ^3^Centre of Excellence Plant and Vegetation Ecology, Department of Biology, University of AntwerpAntwerp, Belgium

**Keywords:** abiotic stress, reactive oxygen species, oxidative damage, antioxidants, future climate, elevated CO_2_, stress mitigation, photorespiration

## Abstract

Elevated atmospheric CO_2_ can stimulate plant growth by providing additional C (fertilization effect), and is observed to mitigate abiotic stress impact. Although, the mechanisms underlying the stress mitigating effect are not yet clear, increased antioxidant defenses, have been held primarily responsible (antioxidant hypothesis). A systematic literature analysis, including “all” papers [Web of Science (WoS)-cited], addressing elevated CO_2_ effects on abiotic stress responses and antioxidants (105 papers), confirms the frequent occurrence of the stress mitigation effect. However, it also demonstrates that, in stress conditions, elevated CO_2_ is reported to increase antioxidants, only in about 22% of the observations (e.g., for polyphenols, peroxidases, superoxide dismutase, monodehydroascorbate reductase). In most observations, under stress and elevated CO_2_ the levels of key antioxidants and antioxidant enzymes are reported to remain unchanged (50%, e.g., ascorbate peroxidase, catalase, ascorbate), or even decreased (28%, e.g., glutathione peroxidase). Moreover, increases in antioxidants are not specific for a species group, growth facility, or stress type. It seems therefore unlikely that increased antioxidant defense is the major mechanism underlying CO_2_-mediated stress impact mitigation. Alternative processes, probably decreasing the oxidative challenge by reducing ROS production (e.g., photorespiration), are therefore likely to play important roles in elevated CO_2_ (relaxation hypothesis). Such parameters are however rarely investigated in connection with abiotic stress relief. Understanding the effect of elevated CO_2_ on plant growth and stress responses is imperative to understand the impact of climate changes on plant productivity.

## Introduction

The changing earth's atmosphere includes a gradual increase in CO_2_ to possibly double the current concentration (IPCC, 2012). Such increase in primary carbon (C) source, will affect plant metabolism, growth, and development (fertilizing effect), especially under favorable water and nutrient conditions. Such effect may be transient, and differ among plant groups, particularly between C3- and C4-type photosynthesis.

This subject is extensively reviewed (e.g., Long et al., 2006; Ainsworth et al., 2008; Albert et al., 2011; Dieleman et al., 2012; Xu et al., 2013, 2015; Huang and Xu, 2015; Pandey et al., 2015; Kimball, 2016), and is commonly covered in plant physiology text books.

Less documented are the interactions of elevated CO_2_ with plant responses to the environment, such as in stress conditions. Nevertheless, the importance of understanding such interactions, given the high-CO_2_ future climate scenario's, is increasingly recognized, (e.g., Mittler and Blumwald, 2010; Feng et al., 2014; Xu et al., 2015), also in text books (e.g., see “stress matrix” in Taiz et al., 2015). One effect of elevated CO_2_ on plant responses, is the reduction of stress impact. This is demonstrated at the plant growth level, but also at the level of cellular oxidative damage (e.g., lipid peroxidation, protein oxidation), and at the level of stress-generated reactive oxygen species (ROS; Geissler et al., 2010; Mishra et al., 2013; Zinta et al., 2014; AbdElgawad et al., 2015).

A reasonable number of papers has reported the stress-reducing effect of elevated CO_2_. Nevertheless, until recently, this topic was rarely covered in reviews. Now, recent overviews of CO_2_ effects in plants, start dedicating attention to this effect (Feng et al., 2014; Misra and Chen, 2015; Xu et al., 2015). It is generally recognized that CO_2_ effects on abiotic stress impact vary considerably, and, that the underlying mechanisms remain elusive. It is clear that, in addition to providing extra C, elevated CO_2_ induces stomatal closing. This improves water use, protecting against drought stress, and helps to explain reduced impact of ozone stress (reduced uptake). However, reduced oxidative damage and ROS levels under elevated CO_2_, probably involves so called non-stomatal factors (Ghannoum, 2009), including metabolic changes. More specifically, increased C availability, possibly resulting in increased supply of defense (antioxidant) molecules, is often held primarily responsible for improved protection against oxidative damage in elevated CO_2_ (antioxidant hypothesis).

This conclusion is indeed supported by studies, showing increased antioxidant levels and/or antioxidant enzyme activities (Lin and Wang, 2002; Geissler et al., 2010; Pintó-Marijuan et al., 2013; Zinta et al., 2014). However, there is a considerable number of reports in which elevated CO_2_ had little or no effect on antioxidants, or even decreased their levels (Erice et al., 2007; Pérez-López et al., 2010; Farfan-Vignolo and Asard, 2012; Mishra et al., 2013). This indicates that the stress-mitigating effect of elevated CO_2_ cannot be universally attributed to increased antioxidant defenses. A key alternative process probably involved in the effect of elevated CO_2_ on oxidative stress, is photorespiration. Elevated CO_2_ promotes carboxylation over oxygenation at rubisco, reducing reactive oxygen species (ROS) formation (relaxation hypothesis; Long and Drake, 1991; Booker et al., 1997; Ainsworth et al., 2008; Zinta et al., 2014; AbdElgawad et al., 2015). Moreover, when measured simultaneously, reduced photorespiration correlates well with the decrease in H_2_O_2_ and lower oxidative damage levels under high CO_2_ in some studies (Aranjuelo et al., 2008; Mishra et al., 2013). Therefore, whether elevated CO_2_ reduces stress impact through “increased defense or decreased challenge,” remains unaddressed (e.g., Tausz-Posch et al., 2013; Xu et al., 2015).

## A systematic literature analysis

To gain insight in this issue, we performed a systematic literature analysis, using an “as complete as possible” collection of studies (Web of Science-indexed), meeting the following criteria; (1) analyzing oxidative stress markers (electrolyte leakage, protein oxidation, lipid peroxidation) and antioxidants (molecules and enzymes), (2) in plant shoots grown under ambient and elevated CO_2_, in presence and absence of abiotic stress. The selection of papers was based on a search using the keyword combination “elevated CO_2_” AND antioxidants OR oxidative stress AND plants, resulting in 238 hits (December 2015). From the combined lists, double references were removed; manuscripts were removed because measurements were not performed on shoots; because they were reviews; or because one of the keywords (elevated CO_2_, antioxidants, oxidative stress) was in fact not studied in detail but occurred only in the extended keyword list. Eventually, 105 papers were analyzed (details in Supplementary Table 1 and Datasheet 1).

To perform the analysis in a quantitative manner, we scored the number of occurrences (observations) in which a given parameter (e.g., lipid peroxidation, ascorbate level, APX activity,…), significantly increased (+), remained unchanged (=), or decreased (−). This number of observations (+ or = or −), is expressed relative, as a fraction (%) of the total number (sum = n) of observations on that parameter. As not all 105 studies report on all parameters, the number of observations is often lower than 105. On the other hand, often papers report on changes in one particular parameter, in multiple measurements and/or conditions, or development stages, with and without elevated CO_2_. Therefore, the number of observations can be considerably higher than 105 (see figures).

Changes (+/ = /−) were recorded for three plant treatments: effect of: (1) elevated CO_2_ relative to ambient CO_2_ (labeled “C” in heat maps); (2) stress exposure in ambient CO_2_ (relative to non-stressed, “S”); (3) stress exposure in elevated CO_2_ (relative to stress exposure in ambient CO_2_, “CS”). These nine sets of observations (+/ = /− for each C/S/CS), are presented in 3 × 3 heat-map format (vertically treatments: C, S, CS; horizontally: +, =, −). Statistical significance of the observations was taken as reported by the authors of the original papers.

The following, commonly quantified parameters were included; (1) cell damage: lipid peroxidation (malondialdehyde, MDA), protein oxidation (carbonylation), electrolyte leakage; (2) molecular and enzymatic antioxidants: phenolics (PHEN), ascorbate (ASC), glutathione (GSH), tocopherol (TOH), superoxide dismutase (SOD), catalase (CAT), peroxidase (POX), ascorbate peroxidase (APX), monodehydroascorbate reductase (MDHAR), dehydroascorbate reductase (DHAR), glutathione peroxidase (GPX), glutathione reductase (GR); (3) reactive oxygen species (ROS): hydrogen peroxide (H_2_O_2_), and superoxide (${\text{O}}_{2}^{-∙}$).

To approximate changes in “*overall* (total) oxidative damage,” and “*overall* (total) antioxidants,” we calculated the mathematical sum of the number of observations (either +, =, or −) of all oxidative damage parameters (lipid peroxidation + protein oxidation + electrolyte leakage), and the sum of the number of changes, in all antioxidant parameters (PHEN + ASC + GSH + TOH + SOD + CAT + POX + APX + MDHAR + DHAR + GPX + GR), respectively. To evaluate if elevated CO_2_ effects on antioxidants were specific for certain species groups, growth facility or stress type, we categorized the observations in, (a) C3/C4 metabolism, (b) species groups [grasses (Poaceae), legumes (Fabaceae), trees], (c) growth facility [growth chamber, green house, open top chambers (OTC), Free Air Concentration Enrichment (FACE)]; and (d) stress type (heavy metals, drought, high temperature, ozone, salinity).

## Elevated CO_2_ mitigates oxidative stress in plants

First we addressed the question whether changes in “overall oxidative damage,” induced by C, S, and CS, correlate with observations of “overall changes in antioxidants.” Such correlation could point to a causal link, e.g., increased antioxidant defenses being responsible for reduced stress impact in elevated CO_2_ (antioxidant hypothesis). Obviously, estimating *overall* levels is a rather crude approach, and does not eliminate the possibility of elevated CO_2_ specifically affecting one or more antioxidant components (analyzed below).

**Figure 1:**
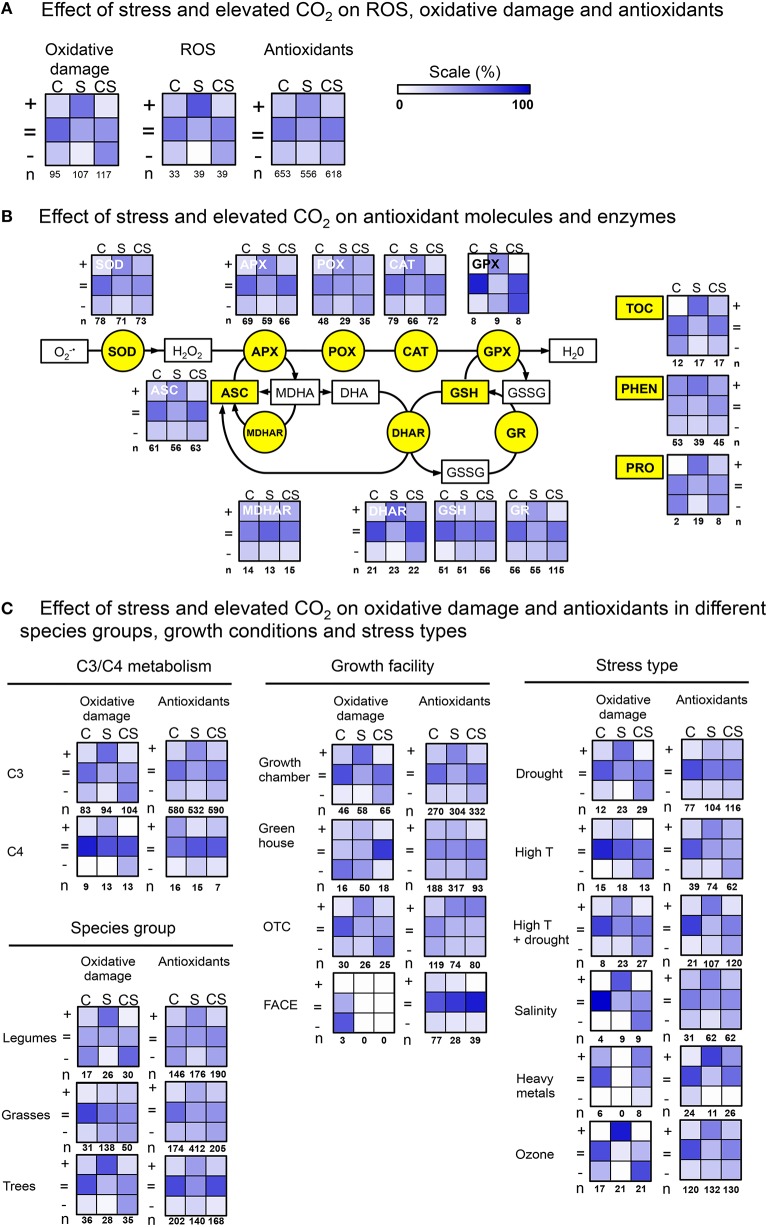
**Heat maps showing the relative numbers of observations (%), demonstrating the effect (increase “+, ”no change “ =, ” decrease “−”) of elevated CO_**2**_ (C), stress (S), and their combination (CS), on oxidative damage and antioxidants. (A)** Comparing overall effects on oxidative damage, ROS and antioxidants. **(B)** Effects individual antioxidant components. **(C)** Effects categorized by metabolism type, species group, growth conditions and stress type.

Changes in overall oxidative damage (Figure 1A), largely confirm previous knowledge. First, elevated CO_2_ alone is not often reported to cause cell damage (17% “+,”lane “C”), it rather leaves damage levels unchanged (60% “ = ”), or, in some instances, decreased (23% “−”). Second, abiotic stress increases cell damage indicators in most observations (55% “+,”lane “S”). Third, elevated CO_2_ is frequently reported to reduce stress impact (47% “−,”lane “CS,” also compare “S”and “CS”in “+”row). This pattern of changes in C, S and CS, correlates very well with the changes in ROS levels (mostly H_2_O_2_; Figure 1A, panel “ROS”). Elevated CO_2_alone (C) was not often observed to increase ROS levels (24% “+”), but rather did not affect (55% “ = ”), or decrease ROS (21% “−”). Stress increased ROS levels at ambient CO_2_ (67% “+”). The correlation between changes in oxidative damage and ROS levels, is perhaps not very surprising, and probably indicates that cell damage under stress is primarily caused by oxidative effects.

However, changes induced by C, S, and CS in “overall antioxidants” (Figure 1A) are different from changes in oxidative damage and ROS. In particular, stress induces increases in antioxidants less frequent (43%, “+”) than increases in oxidative damage (55% “+”) or ROS (67% “+”). And, antioxidants were more often reported to not change (50% “ = ”), then this is the case for oxidative damage (36% “ = ”) and ROS (33% “ = ”) changes. Therefore, it appears questionable whether increases in antioxidants can be considered a likely general cause of lowered ROS and oxidative damage in elevated CO_2_.

## Effect of elevated CO_2_ on individual antioxidant components

It is conceivable that elevated CO_2_ specifically increases particular antioxidants. Such information might be lost in the somewhat “crude” summation of all observations (as done above). We therefore zoomed-in on individual molecules and enzymes from various antioxidant defense pathways (Figure 1B). This analysis confirms reported increases of the activities of many antioxidant components under abiotic stress (lanes S). Increases were particularly frequently observed for PHEN (54% “+”), DHAR (61% “+”), and TOC (59% “+”). However, for all antioxidant components there is a considerable number of observations indicating no changes (from 22 to 62% “ = ”).

In elevated atmospheric CO_2_ during stress exposure, the levels and activities of nearly all molecular and enzymatic antioxidants (except GPX) have been reported to increase, but with variable and often low (10–40%) frequencies. Increases were reported particularly frequently for PHEN (38%), POX (26%), SOD (29%), and MDHAR (40%). None of all antioxidant components accounts solely, throughout all studies, for increased antioxidant defenses in high CO_2_. The frequently observed increases of DHAR and MDHAR, together with ASC and GSH levels reported to remain largely unchanged (56 and 54% “ = ”), may indicate that the ASC/GSH-cycle is often involved in the antioxidant response in elevated CO_2_. However, MDHAR/DHAR activities are reported in only 12 of the 105 papers, and further verification is therefore necessary.

## Are antioxidants specifically responsive to elevated CO_2_ in particular species or growth conditions?

We next addressed the question whether increases in antioxidant capacity in elevated CO_2_ were possibly specific for metabolism type (C3 vs. C4), species-group, stress type or growth facilities (details in Supplementary Table 1). For example, one could hypothesize that in C4 plants, extra CO_2_ is more likely to alleviate stress impact through increased antioxidant capacity, then through suppression of photorespiration. Our dataset contains 54 species with C3, and 8 with C4 metabolism. Heat maps summarizing the observations on oxidative damage and antioxidant changes (Figure 1C), show considerable reduction of stress impact by elevated CO_2_ and increases in antioxidants for C3 as well as C4 plants.

A large number (95%) of all species in which abiotic stress, elevated CO_2_ and antioxidant changes are studied, are either legumes (10), grasses (18), or trees (22) (Supplementary Table 1). Oxidative damage as a result of abiotic stress, was considerably more frequently observed in the legumes (58%) and trees (68%), compared to grasses (17%) (Figure 1C). Also the reduction in oxidative damage by elevated CO_2_, was more frequently reported in legumes (47%), than in grasses (16%) and trees (11%). It is not immediately clear what the basis is for this difference in responsiveness. Increases in antioxidants are reported equally frequent in each species group.

Another factor that could explain some inconsistency in reports on antioxidant increases in elevated CO_2_, is the variation in growth facility. Elevated CO_2_ effects on growth seem less pronounced in FACE experiments, than in growth cabinet-experiments (Ainsworth et al., 2008). On the other hand, in field conditions (e.g., OTC, FACE) stress is often more severe and prolonged (Mittler and Blumwald, 2010). The literature analysis shows that reduction of stress impact on cell damage, and increases in antioxidants, are reported in all facilities (Figure 1C, insufficient data for FACE).

Finally, we also sorted the observations by stress type (Figure 1C). It is apparent that the stress mitigating effect on oxidative damage occurs for all abiotic stresses, with the notable exception of heavy metal stress. However, the effect of elevated CO_2_ on heavy metal stress is only reported in 4/105 papers, and needs further confirmation.

Increases in antioxidants are reported for all stresses, and are therefore not stress-type specific. Interestingly, reported increases in antioxidants for drought and ozone stress, suggest that in addition to stomatal closure by elevated CO_2_, other processes contribute to the reduction of drought and ozone impact. The data sorted by stress type, also illustrate that in the majority of the reports, that whereas antioxidant activities generally increase in response to the stress (S), they do not increase further or even decrease when the stress is combined with elevated CO_2_ (CS).

## Conclusions

The question whether mitigation of stress impact by elevated CO_2_ occurs through “increased defense or decreased challenge” is probably best answered by “both.” Clearly increased defenses have been demonstrated, but only in a minority of the reports, and, this effect is not specific for any particular antioxidant, C3, or C4 metabolism, for a particular species group, growth facility-type or stress type. This suggests that decreased challenge also plays an important role in stress mitigation in elevated CO_2_. The primary candidate process for this, is reduced hydrogen peroxide production by elevated CO_2_ in photorespiration. Effects of elevated CO_2_ on photorespiration in stress conditions, have also been reported, and point to effects on photosynthesis (Booker et al., 1997; Aranjuelo et al., 2008; Pérez-López et al., 2012; AbdElgawad et al., 2015). However, despite the possibly important role of photorespiration in stress responses under future climate conditions, these aspects are rarely investigated simultaneously (only 4 of 105 papers investigated photorespiration changes). It is therefore of great interest to further unravel the role of antioxidants and photorespiration in elevated CO_2_ effects. Moreover, the causal role of antioxidant increases to reduce oxidative damage and ROS under elevated CO_2_, is almost exclusively inferred from “correlative changes.” This conclusion is not, yet, supported by studies performing plant manipulations, e.g., use of mutants, overexpressor lines, or pharmacological treatments. Engineered plants with elevated antioxidant enzymes, sometimes show increased stress resistance (Eltayeb et al., 2007; Lee et al., 2007; Avramova et al., 2015). However, these lines have not been tested under elevated CO_2_. Recently, a mutant screening assay was developed employing the effect of elevated CO_2_ on hydrogen peroxide production (Queval et al., 2012), which underlines the relevance of understanding photorespiration in altered CO_2_ conditions.

**Figure 2:**
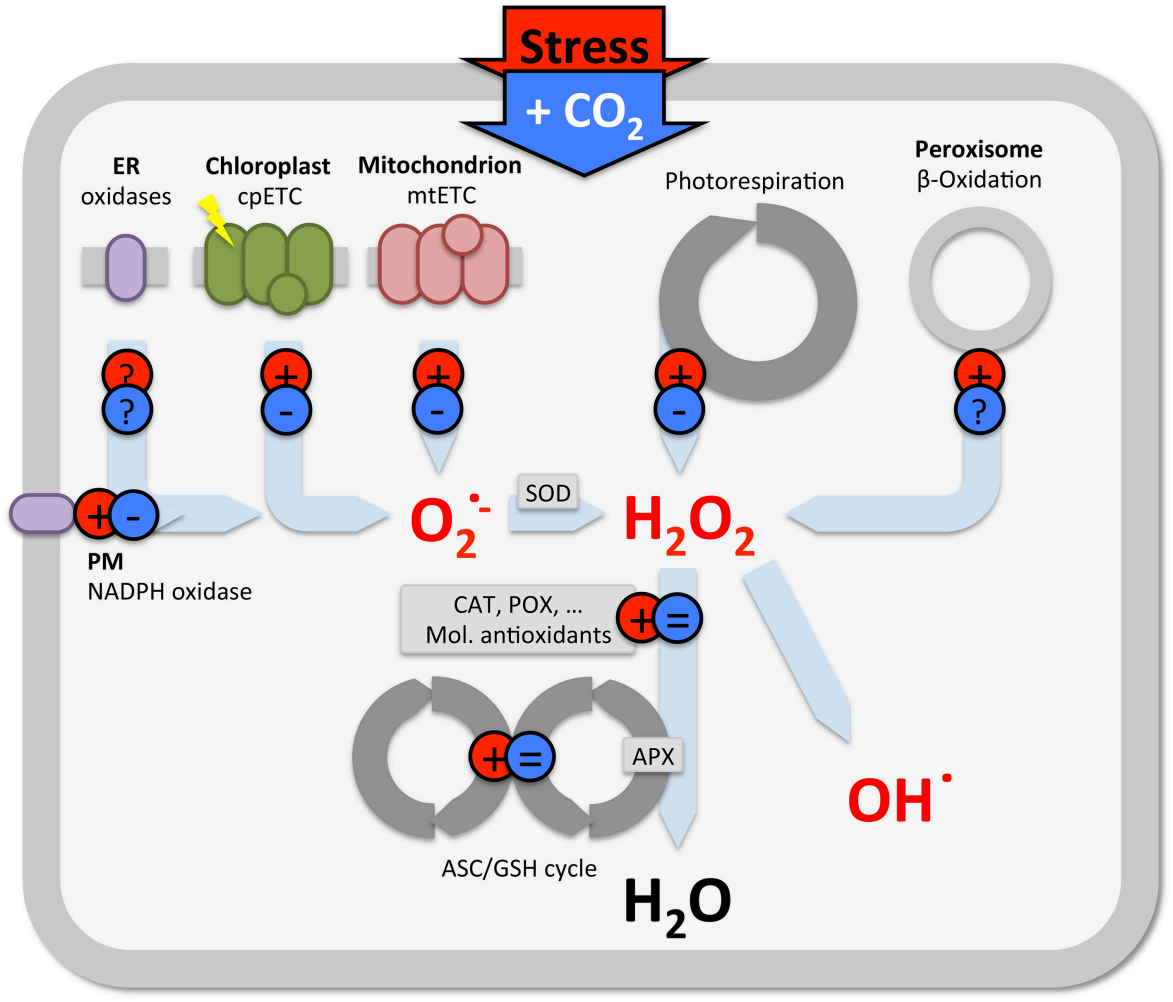
**Schematic view summarizing the predominant effect of stress (red) and elevated CO_**2**_ (blue) on ROS production in various subcellular locations**. Note, there is considerable variety in the outcome of studies on antioxidants (see text), only the predominant effects are indicated in this generalization.

However, also in C4 plants, in which photorespiration is not very active, elevated CO_2_ reduces ROS levels and oxidative damage, without changes in antioxidants. This suggests that other non-stomatal processes, apart from antioxidant defenses and photorespiration, contribute to stress mitigation. Stress induces ROS production at various cellular sites (e.g., Foyer et al., 1994; Schwanz et al., 1996; Gill and Tuteja, 2010; Miller et al., 2010; Das and Roychoudhury, 2014), which each can be affected by elevated CO_2_ (Figure 2). Apart from changes in antioxidants and photorespiration (described above), some evidence indicates that elevated CO_2_ reduces mitochondrial and chloroplast ROS formation, and NADPH oxidase activity, but, little is known at the level of β-oxidation or other cellular oxidases (Gonzalez-Meler et al., 1996; Booker et al., 1997; Gonzàlez-Meler and Siedow, 1999; Lin and Wang, 2002). Understanding the effect of elevated CO_2_ in plant stress responses is further complicated by the fact that additional C affects multiple metabolic processes, as demonstrated by non-targeted (omics) approaches (e.g., Sicher and Barnaby, 2012; Xu et al., 2014; Zinta et al., 2014; AbdElgawad et al., 2015; Misra and Chen, 2015).

An explanation for the relatively large variety in antioxidant responses to elevated CO_2_, is that these responses possibly occur relatively far downstream from the CO_2_ primary targets. As a result, changes in antioxidants may not directly correlate to the stress and CO_2_ treatment only, but are an integrated response of changes in various metabolic processes. The primary targets for CO_2_, i.e., where cellular “perception” of altered CO_2_ levels first occur, are probably changes in stomatal opening, suppression of photorespiration and increased levels of carbohydrates through increased C fixation. Changes in antioxidants are probably an integrated downstream overall result from changes in these processes. It therefore appears that an important topic, as the response of plants to adverse growth conditions, in future climate-levels CO_2_, is underexplored, and leaves many unanswered questions (also Xu et al., 2015).

Finally, with regard to textbook knowledge, our analysis indicates that positive interactions of CO_2_ have been shown for salinity and heat stress. We therefore suggest to update the very instructive Mittler and Blumwald “stress matrix” (Mittler and Blumwald, 2010; Taiz et al., 2015) accordingly.

## Author contributions

HAb: Literature analysis and review writing. GZ: Literature analysis and review writing. GB: Review writing. IJ: Review writing. HA: Literature analysis and review writing.

## Funding

This work was supported by the Research Council of the University of Antwerp as concerted research project (GOA-BOF-UA-2007), and Flemish Science Foundation as concerted research project (FWO, G0D0514N). GZ acknowledges support from Methusalem Funding to the Centre of Excellence “PLECO,” University of Antwerp.

### Conflict of interest statement

The authors declare that the research was conducted in the absence of any commercial or financial relationships that could be construed as a potential conflict of interest.

## Supplementary material

Supplementary Table 1 and Datasheet 1 **Overview of the papers used in this study**.
