# Supplementary material for: Future Climate CO2 Levels Mitigate Stress Impact on Plants: Increased Defense or Decreased Challenge?
Source: Front Plant Sci. 2016 May 2;7:556. doi: 10.3389/fpls.2016.00556 (PMC4852726; doi:10.3389/fpls.2016.00556)
Supplement: Supplementary file 2 [file DataSheet1.docx]

**References for papers listed in Supplementary table 1**

To authors of papers in the same field, that feel their published work meets the criteria (topics) of our publication, and have nevertheless not been included: we apologize. Please contact us and maybe the publisher will allow us an update of supplementary figures and tables, in as much as essential conclusions are not altered.

Abdelgawad, H., Farfan-Vignolo, E.R., De Vos, D., and Asard, H. (2015). Elevated CO_2_ mitigates drought and temperature-induced oxidative stress differently in grasses and legumes. *Plant Science* 231**,** 1-10.

Abdelgawad, H., Peshev, D., Zinta, G., Van Den Ende, W., Janssens, I.A., and Asard, H. (2014). Climate extreme effects on the chemical composition of temperate grassland species under ambient and elevated CO_2_: a comparison of fructan and non-fructan accumulators. *PloS one* 9**,** e92044.

Al-Rawahy, S.H., Sulaiman, H., Farooq, S.A., Karam, M.F., and Sherwani, N. (2013). Effect of O_3_ and CO_2_ Levels on Growth, Biochemical and Nutrient Parameters of Alfalfa (*Medicago Sativa*). *APCBEE Procedia* 5**,** 288-295.

Aranjuelo, I., Erice, G., Nogués, S., Morales, F., Irigoyen, J.J., and Sánchez-Díaz, M. (2008). The mechanism (s) involved in the photoprotection of PSII at elevated CO_2_ in noduƒlated alfalfa plants. *Environmental and experimental botany* 64**,** 295-306.

Bączek-Kwinta, R., and Kościelniak, J. (2009). The mitigating role of environmental factors in seedling injury and chill-dependent depression of catalase activity in maize leaves. *Biologia Plantarum* 53**,** 278-284.

Badiani, M., D'annibale, A., Paolacci, A., Miglietta, F., and Raschi, A. (1993). The antioxidant status of soybean (*Glycine max*) leaves grown under natural CO_2_ enrichment in the field. *Functional Plant Biology* 20**,** 275-284.

Badiani, M., Paolacci, A.R., Fusari, A., Bettarini, I., Brugnoli, E., Lauteri, M., Miglietta, F., and Raschi, A. (1998). Foliar antioxidant status of plants from naturally high‐CO_2_ sites. *Physiologia Plantarum* 104**,** 765-771.

Balouchi, H., Sanavy, S.M., Emam, Y., and Dolatabadian, A. (2009). UV radiation, elevated CO_2_ and water stress effect on growth and photosynthetic characteristics in durum wheat. *Plant, Soil and Environment* 55**,** 443-453.

Bencze, S., Bamberger, Z., Janda, T., Balla, K., Varga, B., Bedő, Z., and Veisz, O. (2014). Physiological response of wheat varieties to elevated atmospheric CO_2_ and low water supply levels. *Photosynthetica* 52**,** 71-82.

Booker, F.L., and Fiscus, E.L. (2005). The role of ozone flux and antioxidants in the suppression of ozone injury by elevated CO_2_ in soybean. *Journal of Experimental Botany* 56**,** 2139-2151.

Burgess, P., and Huang, B. (2014). Root protein metabolism in association with improved root growth and drought tolerance by elevated carbon dioxide in creeping bentgrass. *Field Crops Research* 165**,** 80-91.

Caldwell, C.R., Britz, S.J., and Mirecki, R.M. (2005). Effect of temperature, elevated carbon dioxide, and drought during seed development on the isoflavone content of dwarf soybean [*Glycine max* (L.) Merrill] grown in controlled environments. *Journal of agricultural and food chemistry* 53**,** 1125-1129.

Castells, E., Roumet, C., Penuelas, J., and Roy, J. (2002). Intraspecific variability of phenolic concentrations and their responses to elevated CO_2_ in two Mediterranean perennial grasses. *Environmental and Experimental Botany* 47**,** 205-216.

De Faria, A.P., Fernandes, G.W., and França, M.G.C. (2015). Physiological approaches to determine the impact of climate changes on invasive African grasses in the savanna ecoregion of Brazil. *Environmental Earth Sciences* 74**,** 3077-3088.

De La Mata, L., Cabello, P., De La Haba, P., and Aguera, E. (2012). Growth under elevated atmospheric CO(_2_) concentration accelerates leaf senescence in sunflower (*Helianthus annuus* L.) plants. *J Plant Physiol* 169**,** 1392-1400.

Di Toppi, L.S., Marabottini, R., Badiani, M., and Raschi, A. (2002). Antioxidant status in herbaceous plants growing under elevated CO_2_ in mini-FACE rings. *Journal of plant physiology* 159**,** 1005-1013.

Dickson, R., Coleman, M.D., Riemenschneider, D., Isebrands, J., Hogan, G., and Karnosky, D. (1998). Growth of five hybrid poplar genotypes exposed to interacting elevated CO_2_ and O_3_. *Canadian Journal of Forest Research* 28**,** 1706-1716.

Erice, G., Irigoyen, J.J., Sánchez-Díaz, M., Avice, J.-C., and Ourry, A. (2007). Effect of drought, elevated CO_2_ and temperature on accumulation of N and vegetative storage proteins (VSP) in taproot of nodulated alfalfa before and after cutting. *Plant science* 172**,** 903-912.

Ershova, A., Popova, N., and Berdnikova, O. (2011). Production of reactive oxygen species and antioxidant enzymes of pea and soybean plants under hypoxia and high CO2 concentration in medium. *Russian Journal of Plant Physiology* 58**,** 982-990.

Estiarte, M., Penuelas, J., Kimball, B.A., Hendrix, D.L., Pinter Jr, P.J., Wall, G.W., Lamorte, R.L., and Hunsaker, D.J. (1999). Free‐air CO_2_ enrichment of wheat: leaf flavonoid concentration throughout the growth cycle. *Physiologia Plantarum* 105**,** 423-433.

Farfan-Vignolo, E.R., and Asard, H. (2012). Effect of elevated CO_2_ and temperature on the oxidative stress response to drought in Lolium perenne L. and *Medicago sativa* L. *Plant physiology and biochemistry* 59**,** 55-62.

Faria, T., Vaz, M., Schwanz, P., Polie, A., Pereira, J., and Chaves, M. (1999). Responses of photosynthetic and defence systems to high temperature stress in *Quercus suber* L seedlings grown under elevated CO_2_. *Plant Biology* 1**,** 365-371.

Fu, Y., Shao, L., Liu, H., Li, H., Zhao, Z., Ye, P., Chen, P., and Liu, H. (2015). Unexpected decrease in yield and antioxidants in vegetable at very high CO_2_ levels. *Environmental Chemistry Letters* 13**,** 473-479.

Gao, J.Y., Xu, S., Chen, W., and He, X.Y. (Year). "Response of Antioxidant System in Leaves of Ginkgo biloba to Elevated CO_2_ and/or O_3_ and its Natural Recovery in an Urban Area", in: *Advanced Materials Research*: Trans Tech Publ), 18-21.

Geissler, N., Hussin, S., and Koyro, H.-W. (2009). Elevated atmospheric CO_2_ concentration ameliorates effects of NaCl salinity on photosynthesis and leaf structure of *Aster tripolium* L. *Journal of Experimental Botany* 60**,** 137-151.

Geissler, N., Hussin, S., and Koyro, H.-W. (2010). Elevated atmospheric CO_2_ concentration enhances salinity tolerance in Aster tripolium L. *Planta* 231**,** 583-594.

Ghasemzadeh, A., Jaafar, H.Z., Karimi, E., and Ibrahim, M.H. (2012). Combined effect of CO_2_ enrichment and foliar application of salicylic acid on the production and antioxidant activities of anthocyanin, flavonoids and isoflavonoids from ginger. *BMC complementary and alternative medicine* 12**,** 229.

Ghasemzadeh, A., Jaafar, H.Z., and Rahmat, A. (2010). Elevated carbon dioxide increases contents of flavonoids and phenolic compounds, and antioxidant activities in Malaysian young ginger (*Zingiber officinale* Roscoe.) varieties. *Molecules* 15**,** 7907-7922.

Gillespie, K.M., Xu, F., Richter, K.T., Mcgrath, J.M., Markelz, R.C., Ort, D.R., Leakey, A.D., and Ainsworth, E.A. (2012). Greater antioxidant and respiratory metabolism in field‐grown soybean exposed to elevated O_3_ under both ambient and elevated CO_2_. *Plant, cell & environment* 35**,** 169-184.

Goufo, P., Pereira, J., Figueiredo, N., Oliveira, M.B.P., Carranca, C., Rosa, E.A., and Trindade, H. (2014). Effect of elevated carbon dioxide (CO_2_) on phenolic acids, flavonoids, tocopherols, tocotrienols, γ-oryzanol and antioxidant capacities of rice (*Oryza sativ*a L.). *Journal of Cereal Science* 59**,** 15-24.

Goverde, M., Erhardt, A., and Stöcklin, J. (2004). Genotype-specific response of a lycaenid herbivore to elevated carbon dioxide and phosphorus availability in calcareous grassland. *Oecologia* 139**,** 383-391.

Guo, B., Dai, S., Wang, R., Guo, J., Ding, Y., and Xu, Y. (2015). Combined effects of elevated CO_2_ and Cd-contaminated soil on the growth, gas exchange, antioxidant defense, and Cd accumulation of poplars and willows. *Environmental and Experimental Botany* 115**,** 1-10.

Hoffmann, C., Plocharski, B., Haferkamp, I., Leroch, M., Ewald, R., Bauwe, H., Riemer, J., Herrmann, J.M., and Neuhaus, H.E. (2013). From endoplasmic reticulum to mitochondria: absence of the *Arabidopsis* ATP antiporter endoplasmic Reticulum Adenylate Transporter1 perturbs photorespiration. *Plant Cell* 25**,** 2647-2660.

Ibrahim, M.H., and Jaafar, H.Z. (2011a). Enhancement of leaf gas exchange and primary metabolites under carbon dioxide enrichment up-regulates the production of secondary metabolites in *Labisia pumila* seedlings. *Molecules* 16**,** 3761-3777.

Ibrahim, M.H., and Jaafar, H.Z. (2012). Impact of elevated carbon dioxide on primary, secondary metabolites and antioxidant responses of *Eleais guineensis* Jacq.(Oil Palm) seedlings. *Molecules* 17**,** 5195-5211.

Ibrahim, M.H., Jaafar, H.Z., Rahmat, A., and Rahman, Z.A. (2011). The relationship between phenolics and flavonoids production with total non structural carbohydrate and photosynthetic rate in *Labisia pumila* Benth. under high CO_2_ and nitrogen fertilization. *Molecules* 16**,** 162-174.

Ibrahim, M.H., and Jaafar, H.Z.E. (2011b). Involvement of Carbohydrate, Protein and Phenylanine Ammonia Lyase in Up-Regulation of Secondary Metabolites in *Labisia pumila* under Various CO_2_ and N2 Level. *Molecules* 16**,** 4172-4190.

Jaafar, H.Z., Ibrahim, M.H., and Karimi, E. (2012). Phenolics and flavonoids compounds, phenylanine ammonia Lyase and antioxidant activity responses to elevated CO_2_ in *Labisia pumila* (Myrisinaceae). *Molecules* 17**,** 6331-6347.

Jia, Y., Tang, S., Wang, R., Ju, X., Ding, Y., Tu, S., and Smith, D.L. (2010). Effects of elevated CO_2_ on growth, photosynthesis, elemental composition, antioxidant level, and phytochelatin concentration in *Lolium mutiforum* and *Lolium perenne* under Cd stress. *Journal of hazardous materials* 180**,** 384-394.

Johnson, R., and Lincoln, D. (1990). Sagebrush and grasshopper responses to atmospheric carbon dioxide concentration. *Oecologia* 84**,** 103-110.

Kobayakawa, H., and Imai, K. (2011). Effects of the interaction between ozone and carbon dioxide on gas exchange, photosystem II and antioxidants in rice leaves. *Photosynthetica* 49**,** 227-238.

Koti, S., Reddy, K.R., Kakani, V., Zhao, D., and Gao, W. (2007). Effects of carbon dioxide, temperature and ultraviolet-B radiation and their interactions on soybean (*Glycine max* L.) growth and development. *Environmental and Experimental Botany* 60**,** 1-10.

Kumari, S., Agrawal, M., and Singh, A. (2015). Effects of ambient and elevated CO_2_ and ozone on physiological characteristics, antioxidative defense system and metabolites of potato in relation to ozone flux. *Environmental and Experimental Botany* 109**,** 276-287.

Kumari, S., Agrawal, M., and Tiwari, S. (2013). Impact of elevated CO_2_ and elevated O_3_ on *Beta vulgaris* L.: pigments, metabolites, antioxidants, growth and yield. *Environ Pollut* 174**,** 279-288.

Kuokkanen, K., Niemelä, P., Matala, J., Julkunen‐Tiitto, R., Heinonen, J., Rousi, M., Henttonen, H., Tahvanainen, J., and Kellomäki, S. (2004). The effects of elevated CO_2_ and temperature on the resistance of winter‐dormant birch seedlings (*Betula pendula*) to hares and voles. *Global Change Biology* 10**,** 1504-1512.

Lambreva, M., Christov, K., and Tsonev, T. (2006). Short-term effect of elevated CO_2_ concentration and high irradiance on the antioxidant enzymes in bean plants. *Biologia plantarum* 50**,** 617-623.

Lee, S.H., Woo, S.Y., and Je, S.M. (2015). Effects of elevated CO_2_ and water stress on physiological responses of Perilla frutescens var. japonica HARA. *Plant Growth Regulation* 75**,** 427-434.

Levine, L.H., and Paré, P.W. (2009). Antioxidant capacity reduced in scallions grown under elevated CO_2_ independent of assayed light intensity. *Advances in Space Research* 44**,** 887-894.

Li, D., Liu, H., Qiao, Y., Wang, Y., Dong, B., Cai, Z., Shi, C., Liu, Y., Li, X., and Liu, M. (2013a). Physiological regulation of soybean (*Glycine ma*x L. Merr.) growth in response to drought under elevated CO_2_. *Journal of Food, Agriculture & Environment* 11**,** 649-654.

Li, L., Zhang, Y., Luo, J., Korpelainen, H., and Li, C. (2013b). Sex-specific responses of *Populus yunnanensis* exposed to elevated CO_2_ and salinity. *Physiol Plant* 147**,** 477-488.

Li, Q., Liu, B., and Zou, Z. (2012). Effects of Doubled CO_2_ Concentration on Physiological Characteristics of Cucumber Seedlings under Drought Stresses. *Acta Horticulturae***,** 103.

Li, X., Ahammed, G.J., Zhang, Y.Q., Zhang, G.Q., Sun, Z.H., Zhou, J., Zhou, Y.H., Xia, X.J., Yu, J.Q., and Shi, K. (2015). Carbon dioxide enrichment alleviates heat stress by improving cellular redox homeostasis through an ABA-independent process in tomato plants. *Plant Biol (Stuttg)* 17**,** 81-89.

Lin, J.-S., and Wang, G.-X. (2002). Doubled CO_2_ could improve the drought tolerance better in sensitive cultivars than in tolerant cultivars in spring wheat. *Plant Science* 163**,** 627-637.

Liu, L., King, J.S., and Giardina, C.P. (2005). Effects of elevated concentrations of atmospheric CO_2_ and tropospheric O_3_ on leaf litter production and chemistry in trembling aspen and paper birch communities. *Tree Physiology* 25**,** 1511-1522.

Lu, T., He, X., Chen, W., Yan, K., and Zhao, T. (2009). Effects of elevated O_3_ and/or elevated CO_2_ on lipid peroxidation and antioxidant systems in *Ginkgo biloba* leaves. *Bulletin of environmental contamination and toxicology* 83**,** 92-96.

Mamatha, H., Rao, N.S., Laxman, R., Shivashankara, K., Bhatt, R., and Pavithra, K. (2014). Impact of elevated CO_2_ on growth, physiology, yield, and quality of tomato (*Lycopersicon esculentum* Mill) cv. Arka Ashish. *Photosynthetica* 52**,** 519-528.

Marabottini, R., Schraml, C., Paolacci, A., Raschi, A., Rennenberg, H., and Badiani, M. (2001). Foliar antioxidant status of adult Mediterranean oak species (*Quercus ilex* L. and *Q. pubescens* Willd.) exposed to permanent CO_2_-enrichment and to seasonal water stress. *Environmental Pollution* 115**,** 413-423.

Mckee, I., Bullimore, J., and Long, S. (1997). Will elevated CO_2_ concentrations protect the yield of wheat from O_3_ damage? *Plant, Cell & Environment* 20**,** 77-84.

Mishra, A.K., Rai, R., and Agrawal, S. (2013). Individual and interactive effects of elevated carbon dioxide and ozone on tropical wheat (*Triticum aestivum* L.) cultivars with special emphasis on ROS generation and activation of antioxidant defence system. *Indian J Biochem Biophys* 50**,** 139-149.

Mishra, S., Heckathorn, S.A., Barua, D., Wang, D., Joshi, P., Hamilton Iii, E.W., and Frantz, J. (2008). Interactive Effects of Elevated CO_2_ and Ozone on Leaf Thermotolerance in Field‐grown Glycine max. *Journal of integrative plant biology* 50**,** 1396-1405.

Naudts, K., Van Den Berge, J., Farfan, E., Rose, P., Abdelgawad, H., Ceulemans, R., Janssens, I., Asard, H., and Nijs, I. (2014). Future climate alleviates stress impact on grassland productivity through altered antioxidant capacity. *Environmental and Experimental Botany* 99**,** 150-158.

Niewiadomska, E., Gaucher-Veilleux, C., Chevrier, N., Mauffette, Y., and Dizengremel, P. (1999). Elevated CO_2_ Does not Provide Protection against Ozone Considering the Activity of Several Antioxidant Enzymes in the Leaves of Sugar Maple. *Journal of Plant Physiology* 155**,** 70-77.

O’neill, B.F., Zangerl, A.R., Dermody, O., Bilgin, D.D., Casteel, C.L., Zavala, J.A., Delucia, E.H., and Berenbaum, M.R. (2010). Impact of elevated levels of atmospheric CO_2_ and herbivory on flavonoids of soybean (*Glycine max* Linnaeus). *Journal of chemical ecology* 36**,** 35-45.

Oksanen, E., Riikonen, J., Kaakinen, S., Holopainen, T., and Vapaavuori, E. (2005). Structural characteristics and chemical composition of birch (Betula pendula) leaves are modified by increasing CO_2_ and ozone. *Global Change Biology* 11**,** 732-748.

Padu, E., Kollist, H., Tulva, I., Oksanen, E., and Moldau, H. (2005). Components of apoplastic ascorbate use in Betula pendula leaves exposed to CO_2_ and O_3_ enrichment. *New phytologist* 165**,** 131-142.

Penuelas, J., Estiarte, M., and Llusia, J. (1997). Carbon-based secondary compounds at elevated CO_2_. *Photosynthetica* 33**,** 313-319.

Pérez-López, U., Miranda-Apodaca, J., Lacuesta, M., Mena-Petite, A., and Muñoz-Rueda, A. (2015a). Growth and nutritional quality improvement in two differently pigmented lettuce cultivars grown under elevated CO_2_ and/or salinity. *Scientia Horticulturae* 195**,** 56-66.

Pérez-López, U., Miranda-Apodaca, J., Muñoz-Rueda, A., and Mena-Petite, A. (2013). Lettuce production and antioxidant capacity are differentially modified by salt stress and light intensity under ambient and elevated CO_2_. *Journal of plant physiology* 170**,** 1517-1525.

Pérez-López, U., Miranda-Apodaca, J., Muñoz-Rueda, A., and Mena-Petite, A. (2015b). Interacting effects of high light and elevated CO_2_ on the nutraceutical quality of two differently pigmented *Lactuca sativa* cultivars (Blonde of Paris Batavia and Oak Leaf). *Scientia Horticulturae* 191**,** 38-48.

Pérez‐López, U., Robredo, A., Lacuesta, M., Sgherri, C., Mena‐Petite, A., Navari‐Izzo, F., and Muñoz‐Rueda, A. (2010). Lipoic acid and redox status in barley plants subjected to salinity and elevated CO_2_. *Physiologia plantarum* 139**,** 256-268.

Pérez‐López, U., Robredo, A., Lacuesta, M., Sgherri, C., Muñoz‐Rueda, A., Navari‐Izzo, F., and Mena‐Petite, A. (2009). The oxidative stress caused by salinity in two barley cultivars is mitigated by elevated CO_2_. *Physiologia Plantarum* 135**,** 29-42.

Pietrini, F., Bianconi, D., Massacci, A. and. Iannelli M.A. (2016). Combined effects of elevated CO_2_ and Cd-contaminated water on growth, photosynthetic response, Cd accumulation and thiolic components status in *Lemna minor* L. *Journal of Hazardous Materials* 309,77-86.

Pintó‐Marijuan, M., Joffre, R., Casals, I., De Agazio, M., Zacchini, M., García‐Plazaola, J.I., Esteban, R., Aranda, X., Guàrdia, M., and Fleck, I. (2013). Antioxidant and photoprotective responses to elevated CO_2_ and heat stress during holm oak regeneration by resprouting, evaluated with NIRS (near‐infrared reflectance spectroscopy). *Plant Biology* 15**,** 5-17.

Polle, A., Eiblmeier, M., Sheppard, L., and Murray, M. (1997). Responses of antioxidative enzymes to elevated CO_2_ in leaves of beech (*Fagus sylvatica* L.) seedlings grown under a range of nutrient regimes. *Plant, Cell and Environment* 20**,** 1317-1321.

Polle, A., Pfirrmann T., Chakrabarti, S., and Rennenberg, H. (1993) The effects of enhanced ozone and enhanced carbon dioxide concentrations on biomass, pigments and antioxidative enzymes in spruce needles (*Picea abies* L.). *Plant, Cell and Environment* 16, 311–316.

Pritchard, S.G., Ju, Z., Van Santen, E., Qiu, J., Weaver, D.B., Prior, S.A., and Rogers, H.H. (2000). The influence of elevated CO_2_ on the activities of antioxidative enzymes in two soybean genotypes. *Functional Plant Biology* 27**,** 1061-1068.

Rao, M.V., Hale, B.A., and Ormrod, D.P. (1995). Amelioration of ozone-induced oxidative damage in Wheat plants grown under high carbon dioxide (Role of antioxidant enzymes). *Plant Physiology* 109**,** 421-432.

Reddy, G.V., Tossavainen, P., Nerg, A.-M., and Holopainen, J.K. (2004). Elevated atmospheric CO_2_ affects the chemical quality of Brassica plants and the growth rate of the specialist, *Plutella xylostella*, but not the generalist, Spodoptera littoralis. *Journal of agricultural and food chemistry* 52**,** 4185-4191.

Ren, J., Guo, S., Xu, C., Yang, C., Ai, W., Tang, Y., and Qin, L. (2014). Effects of different carbon dioxide and LED lighting levels on the anti-oxidative capabilities of Gynura bicolor DC. *Advances in Space Research* 53**,** 353-361.

Robinson, J.M., and Sicher, R.C. (2004). Antioxidant Levels Decrease in Primary Leaves of Barley during Growth at Ambient and Elevated Carbon Dioxide Levels1. *International Journal of Plant Sciences* 165**,** 965-972.

Sakalauskienė, S., Sakalauskaitė, J., Lazauskas, S., Povilaitis, V., Auškalnienė, O., Pšibišauskienė, G., Samuolienė, G., Brazaitytė, A., and Duchovskis, P. (2013). Interactive effects of elevated CO. *Journal of Food, Agriculture & Environment* 11**,** 1121-1124.

Salazar‐Parra, C., Aguirreolea, J., Sánchez‐Díaz, M., Irigoyen, J.J., and Morales, F. (2012). Climate change (elevated CO_2_, elevated temperature and moderate drought) triggers the antioxidant enzymes' response of grapevine cv. Tempranillo, avoiding oxidative damage. *Physiologia plantarum* 144**,** 99-110.

Schwanz, P., Picon, C., Vivin, P., Dreyer, E., Guehl, J.-M., and Polle, A. (1996a). Responses of antioxidative systems to drought stress in pendunculate oak and maritime pine as modulated by elevated CO_2_. *Plant Physiology* 110**,** 393-402.

Schwanz P., Häberle, K.H. and Polle, A. (1996b) Interactive effects of elevated CO_2,_ ozone and drought stress on the activities of antioxidative enzymes in needles of Norway spruce trees (*Picea abies* [L.] Karsten) grown with luxurious N-supply. Journal of Plant Physiology 148, 351–355.

Schwanz, P., and Polle, A. (2001). Differential stress responses of antioxidative systems to drought in pendunculate oak (*Quercus robur*) and maritime pine (*Pinus pinaster*) grown under high CO_2_ concentrations. *Journal of Experimental Botany* 52**,** 133-143.

Sgherri, C.L., Salvateci, P., Menconi, M., Raschi, A., and Navari-Izzo, F. (2000). Interaction between drought and elevated CO_2_ in the response of alfalfa plants to oxidative stress. *Journal of plant physiology* 156**,** 360-366.

Singh, A., and Agrawal, M. (2015). Effects of ambient and elevated CO_2_ on growth, chlorophyll fluorescence, photosynthetic pigments, antioxidants, and secondary metabolites of *Catharanthus roseus* (L.) G Don. grown under three different soil N levels. *Environmental Science and Pollution Research* 22**,** 3936-3946.

Sirtautas, R., Samuoliene, G., Brazaityte, A., Sakalauskaite, J., Sakalauskiene, S., Virsile, A., Jankauskiene, J., Vastakaite, V., and Duchovskis, P. (2014). Impact of CO_2_ on quality of baby lettuce grown under optimized light spectrum. *Acta Scientiarum Polonorum. Hortorum Cultus* 13:109-118.

Sun, L., Ren, L., Peng, T., Xin, L., Qian, H., and Fu, Z. (2015). Carbon dioxide enrichment and brassinosteroid pretreatment alleviate chlorpyrifos phytotoxicity under suboptimal light and temperature conditions in tomato. *Scientia Horticulturae* 192**,** 256-263.

Sunoj, V., Kumar, S.N., Muralikrishna, K., and Padmanabhan, S. (2015). Enzyme Activities and Nutrient Status in Coconut (*Cocos nucifera* L.) Seedling Rhizosphere Soil after Exposure to Elevated CO_2_ and Temperature. *Journal of the Indian Society of Soil Science* 63**,** 191-199.

Synková, H., and Pospíšilová, J. (2002). In vitro precultivation of tobacco affects the response of antioxidative enzymes to ex vitro acclimation. *Journal of Plant Physiology* 159**,** 781-789.

Takagi, M., El-Shemy, H.A., Sasaki, S., Toyama, S., Kanai, S., Saneoka, H., and Fujita, K. (2009). Elevated CO_2_ concentration alleviates salinity stress in tomato plant. *Acta Agriculturae Scandinavica Section B–Soil and Plant Science* 59**,** 87-96.

Tang, S., Xi, L., Zheng, J., and Li, H. (2003). Response to elevated CO_2_ of Indian mustard and sunflower growing on copper contaminated soil. *Bulletin of environmental contamination and toxicology* 71**,** 988-997.

Tausz-Posch, S., Borowiak, K., Dempsey, R.W., Norton, R.M., Seneweera, S., Fitzgerald, G.J., and Tausz, M. (2013). The effect of elevated CO_2_ on photochemistry and antioxidative defence capacity in wheat depends on environmental growing conditions–A FACE study. *Environmental and Experimental Botany* 88**,** 81-92.

Vurro, E., Bruni, R., Bianchi, A., and Di Toppi, L.S. (2009). Elevated atmospheric CO_2_ decreases oxidative stress and increases essential oil yield in leaves of *Thymus vulgaris* grown in a mini-FACE system. *Environmental and Experimental Botany* 65**,** 99-106.

Wang, M., Dong, C., Fu, Y., and Liu, H. (2015). Growth, morphological and photosynthetic characteristics, antioxidant capacity, biomass yield and water use efficiency of *Gynura bicolor* DC exposed to super-elevated CO_2_. *Acta Astronautica* 114**,** 138-146.

Wustman, B., Oksanen, E., Karnosky, D., Noormets, A., Isebrands, J., Pregitzer, K., Hendrey, G., Sober, J., and Podila, G. (2001). Effects of elevated CO_2_ and O_3_ on aspen clones varying in O_3_ sensitivity: can CO_2_ ameliorate the harmful effects of O_3_? *Environmental Pollution* 115**,** 473-481.

Wustman, B., Oksanen, E., Karnosky, D., Noormets, A., Isebrands, J., Pregitzer, K., Hendrey, G., Sober, J., and Podila, G. (2003). Effects of elevated CO_2_ and O_3_ on aspen clones of varying O_3_ sensitivity. *Developments in Environmental Science* 3**,** 391-409.

Xu, S., He, X., Chen, W., Su, D., and Huang, Y. (2014a). Elevated CO_2_ ameliorated the adverse effect of elevated O_3_ in previous-year and current-year needles of Pinus tabulaeformis in urban area. *Bulletin of environmental contamination and toxicology* 92**,** 733-737.

Xu, Z., Shimizu, H., Ito, S., Yagasaki, Y., Zou, C., Zhou, G., and Zheng, Y. (2014b). Effects of elevated CO_2_, warming and precipitation change on plant growth, photosynthesis and peroxidation in dominant species from North China grassland. *Planta* 239**,** 421-435.

Yan, K., Chen, W., Zhang, G., Xu, S., Liu, Z., He, X., and Wang, L. (2010). Elevated CO_2_ ameliorated oxidative stress induced by elevated O_3_ in Quercus mongolica. *Acta physiologiae plantarum* 32**,** 375-385.

Yu, J., Yang, Z., Jespersen, D., and Huang, B. (2014). Photosynthesis and protein metabolism associated with elevated CO_2_-mitigation of heat stress damages in tall fescue. *Environmental and Experimental Botany* 99**,** 75-85.

Zaghdoud, C., Carvajal, M., Moreno, D. Á, Ferchichi, A., and Martínez-Ballesta, M. C. (2016). Health-promoting compounds of broccoli (*Brassica oleracea*L. var. italica) plants as affected by nitrogen fertilisation in projected future climatic change environments. *Journal of the Science of Food and Agriculture*. 96, [392-403](http://dx.doi.org/10.1002/jsfa.7102).

Zhang, F.-F., Wang, Y.-L., Huang, Z.-Z., Zhu, X.-C., Zhang, F.-J., Chen, F.-D., Fang, W.-M., and Teng, N.-J. (2012). Effects of CO_2_ Enrichment on Growth and Development of Impatiens hawkeri. *The Scientific World Journal* 2012.

Zinta, G., Abdelgawad, H., Domagalska, M.A., Vergauwen, L., Knapen, D., Nijs, I., Janssens, I.A., Beemster, G.T., and Asard, H. (2014). Physiological, biochemical, and genome‐wide transcriptional analysis reveals that elevated CO_2_ mitigates the impact of combined heat wave and drought stress in *Arabidopsis thaliana* at multiple organizational levels. *Global change biology* 20**,** 3670-3685.
